# Characterization of the complete mitochondrial DNA sequence of the *Lagocephalus gloveri* (Tetraodontidae, Tetraodontiformes)

**DOI:** 10.1080/23802359.2020.1832933

**Published:** 2020-11-03

**Authors:** Xuanyun Huang, Yongfu Shi, Xiaosheng Shen, Dongmei Huang, Yuan Wang, Jiajie Chen, Youqiong Cai

**Affiliations:** Key Laboratory of East China Sea Fishery Resources Exploitation, Ministry of Agriculture and Rural Affairs, East China Sea Fisheries Research Institute, Chinese Academy of Fishery Sciences, Shanghai, China

**Keywords:** Complete mitochondria genome, *Lagocephalus gloveri*, Tetraodontidae, phylogenetic analysis

## Abstract

The complete mitochondrial genome of *Lagocephalus gloveri* is reported in the present study, which is 16,446 bp in length. It consists of 13 protein-coding genes, two ribosomal RNA genes, 22 transfer RNA genes and a non-coding control region. The overall base composition of the genome is 27.58% for A, 25.07% for T, 30.83% for C and 16.52% for G. The phylogenetic tree, which is based on 12 protein coding gene sequences, suggested that *L. gloveri* was closest to *L. lagocephalus*. This study could give impetus to studies focused on population structure and molecular evolution of *L. gloveri*.

*Lagocephalus gloveri* (Clarke 1897), a senior synonym of *Lagocephalus cheesemanii* (Matsuura and Satoh 2017), belonged to the family Tetraodontidae, subfamily Tetraodontinae. This species was distributed in the Indo-West Pacific, from Japan to South China Sea (Abe and Tabeta 1983). The complete mitochondrial genome of *L. gloveri* was reported and characterized herein, which we hope to provide significant information for further studies on its taxonomy and population genetics.

The sample of *L. gloveri* was collected from Baimajing dock, Zhanzhou city, Hainan province, China. DNA materials were extracted from muscle tissues using the TIANamp Marine Animals DNA Kit (TIANGEN Biotech, Beijing, China). The complete mitochondrial genome was obtained through *de novo* assembly of 1.2 Gb Illumina Hiseq data in PE150 mode.

The mitochondrial genome DNA of *L. gloveri* was a circular molecular of 16,446 bp in length with a predicted control region of 823 bp in length. In addition, this genome contains 13 protein coding genes (PCGs), 22 transfer RNA (tRNAs), and two ribosomal RNA (rRNAs). Among the 37 genes, 28 were encoded by heavy strand, and the other 9 were encoded by light strand, which were similar with other teleosts. The gene arrangement of the mitogenome was the same with other species in genus *Lagocephalus* (Jiang et al. 2018; Xu et al. 2019). The nucleotide composition is 27.58% for A, 25.07% for T, 30.83% for C and 16.52% for G.

To examine the phylogenetic status of *L. gloveri*, 12 protein coding genes except for ND6 of 13 Tetraodontidae species and one *Benthosema pterotum* as the outgroup, were aligned by the MAFFT version 7 software (Katoh and Standley 2013) (Figure 1). Phylogenetic analysis was conducted based on maximum likelihood (ML) analyses implemented in IQ-TREE 1.5.5 (Nguyen et al., 2015) under the default model. Support for the inferred ML tree was inferred by bootstrapping with 1000 replicates. It revealed that *L. gloveri* had the closest relationship with *L. lagocephalus*.

**Figure 1.**
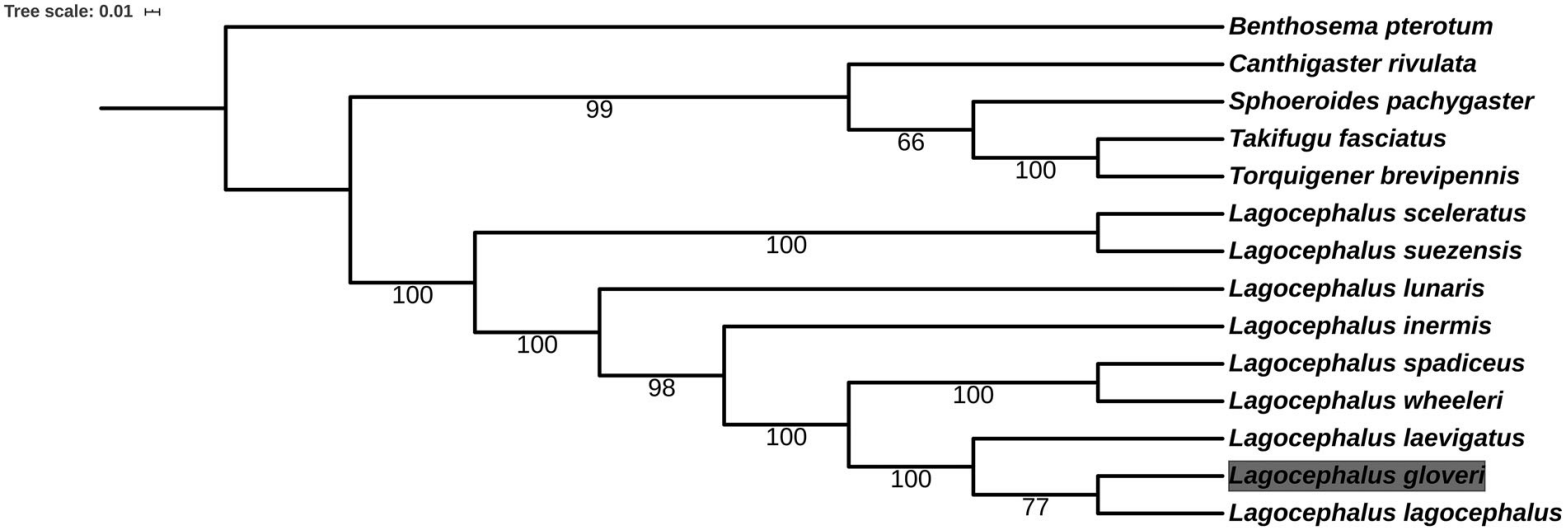
Phylogenetic relationships among 13 Tetraodontidae species and one *Benthosema pterotum* as the outgroup based on 12 PCGs. Bootstrap support values are given at the nodes. Mitochondrial genome accession number used in this phylogeny analysis: *L*agocephalus *sceleratus* MH550879.1; *L. lunaris* GQ461750.1; *L. lagocephalus* AP011933.1; *L. spadiceus* NC_026194.2; *L. suezensis* NC_026229.1; *L. inermis* NC_029376.1; *L. wheeleri* AP009538.1; *L. laevigatus* NC_015345.1; *Takifugu fasciatus* NC_032400.1; *Sphoeroides pachygaster* AP006745.1; *Torquigener brevipennis* AP009537.1; *Canthigaster rivulata* AP006744.1; *Benthosema pterotum* NC_047480.1.

## Data Availability

The data that support the findings of this study are openly available in GenBank of NCBI at https://www.ncbi.nlm.nih.gov, reference number MT903226.
